# *OvMark*: a user-friendly system for the identification of prognostic biomarkers in publically available ovarian cancer gene expression datasets

**DOI:** 10.1186/1476-4598-13-241

**Published:** 2014-10-24

**Authors:** Stephen F Madden, Colin Clarke, Britta Stordal, Mark S Carey, Russell Broaddus, William M Gallagher, John Crown, Gordon B Mills, Bryan T Hennessy

**Affiliations:** Molecular Therapeutics for Cancer Ireland, National Institute for Cellular Biotechnology, Dublin City University, Glasnevin, Dublin 9, Ireland; UCD School of Biomolecular and Biomedical Science, UCD Conway Institute, University College Dublin, Dublin 4, Ireland; Department of Histopathology, St James’ Hospital and Trinity College Dublin, Dublin 8, Ireland; Department of Medical Oncology, Beaumont Hospital, Beaumont Road, P.O. Box 1297, Dublin 9, Ireland; Department of Systems Biology, The University of Texas MD Anderson Cancer Center, Houston, Tx USA; Department of Pathology, The University of Texas MD Anderson Cancer Center, Houston, Tx USA; Department of Obstetrics and Gynecology, University of British Columbia, Vancouver, BC Canada

## Abstract

**Background:**

Ovarian cancer has the lowest survival rate of all gynaecologic cancers and is characterised by a lack of early symptoms and frequent late stage diagnosis. There is a paucity of robust molecular markers that are independent of and complementary to clinical parameters such as disease stage and tumour grade.

**Methods:**

We have developed a user-friendly, web-based system to evaluate the association of genes/miRNAs with outcome in ovarian cancer. The *OvMark* algorithm combines data from multiple microarray platforms (including probesets targeting miRNAs) and correlates them with clinical parameters (e.g. tumour grade, stage) and outcomes (disease free survival (DFS), overall survival). In total, *OvMark* combines 14 datasets from 7 different array platforms measuring the expression of ~17,000 genes and 341 miRNAs across 2,129 ovarian cancer samples.

**Results:**

To demonstrate the utility of the system we confirmed the prognostic ability of 14 genes and 2 miRNAs known to play a role in ovarian cancer. Of these genes, CXCL12 was the most significant predictor of DFS (HR = 1.42, p-value = 2.42x10^−6^). Surprisingly, those genes found to have the greatest correlation with outcome have not been heavily studied in ovarian cancer, or in some cases in any cancer. For instance, the three genes with the greatest association with survival are SNAI3, VWA3A and DNAH12.

**Conclusions/Impact:**

*OvMark* is a powerful tool for examining putative gene/miRNA prognostic biomarkers in ovarian cancer (available at http://glados.ucd.ie/OvMark/index.html). The impact of this tool will be in the preliminary assessment of putative biomarkers in ovarian cancer, particularly for research groups with limited bioinformatics facilities.

**Electronic supplementary material:**

The online version of this article (doi:10.1186/1476-4598-13-241) contains supplementary material, which is available to authorized users.

## Background

Ovarian cancer is the most lethal gynecological malignancy. Due to its lack of early symptoms, this disease is usually diagnosed at an advanced stage when the cancer has already spread to secondary sites [1]. While initial rates of response to first treatment are >80%, the long-term survival rate of patients is very low, mainly due to development of drug resistance [1]. Clinical parameters such as disease stage and tumor grade are important considerations in the management of patients after their initial surgery to establish the necessity for chemotherapy [2]. The amount of residual tumour after surgery is another very important determinant of prognosis. However, reliable markers that are independent of and complementary to clinical parameters are needed for better prognostic stratification of patients and for individualisation of therapy.

For several years now, efforts to identify novel prognostic and predictive factors have focused on molecular markers, with a large number having been identified and investigated [3]. However, while there is evidence that BRCA mutations correlating with the selection of patients for treatment with PARP inhibitors, known ovarian cancer biomarkers are not sufficiently prognostic or predictive, at least for clinical use [3, 4]. It is clear that the selection of molecular markers could benefit greatly from the integration of datasets across multiple studies to increase confidence in the selected markers, by substantially improving the statistical power and robustness of the analysis. To this end, we have developed an easy-to-use algorithm (called *OvMark*) which allows the association of gene(s) of interest with patient outcomes in ovarian cancer. This algorithm integrates gene expression data from 2,129 patients in 14 DNA microarray studies and corresponding clinical (stage, grade, amount of residual disease after surgery, outcomes etc.) and treatment (chemotherapy) data. Among all ovarian cancers in included in *OvMark*, the vast majority are grade 2/3 (high-grade) serous cancers, which compose more than 80% of ovarian cancers that occur in women. The other ovarian cancers in *OvMark* represent less common epithelial ovarian cancer subtypes that are biologically distinct from high-grade serous ovarian cancers, mainly grade 1 (low-grade) serous cancers and low and high-grade endometrioid ovarian cancers. The user can stratify samples based on subtype and these clinical data for investigation of prognostic markers in the context of disease-free survival (DFS) and overall survival (OS). A similar approach has been “previously used” in integrating gene expression and detailed clinical data including survival information in breast cancer [5, 6].

The association of small non-coding RNAs known as microRNAs (miRNAs) with ovarian cancer has been well established [7]. Over the last decade, our understanding of the function that miRNAs play in ovarian and other cancers as well as an array of fundamental biological processes in both plants and animals has increased dramatically [8]. These short endogenous non-coding RNAs act primarily by negatively regulating the expression of target mRNAs through translational inhibition and/or mRNA degradation [8]. Approximately 50% of known human miRNAs are intronic (miRBase release 20, June 2013). Of these, 341 or roughly one third of human miRNA host genes are hybridized by probes on the U133plus2 affymetrix gene chip. A number of studies have reported that many intronic miRNAs show significantly correlated expression profiles with their host genes [9, 10]. Estimates of the number of miRNAs whose expression profiles are significantly correlated with their host gene are as high as 70% [11]. The expression of miRNAs can, in some instances, be inferred from the expression of their host genes, and we have therefore included these miRNAs in our algorithm *OvMark* to allow their evaluation as putative prognostic markers in ovarian cancer from gene expression data.

We confirm the utility of this approach following comparisons of outputs with a panel of 14 previously identified markers of prognosis in ovarian cancer. In addition, we also conducted an unbiased analysis of all genes present in the dataset to assess their prognostic potential and found that the list of the most significant genes is dominated by novel markers of prognosis in ovarian cancer. The feasibility of using miRNA host gene expression as a surrogate for miRNA levels was confirmed using the known miRNA prognostic markers, let-7f-2 [12] and miR-16-1 [13]. Although these markers were previously examined in smaller patient cohorts, *OvMark* was able to confirm the robustness of these prognostic marker across a large and diverse patient dataset.

Our novel user-friendly algorithm *OvMark* is a powerful tool for examining putative gene/miRNA prognostic biomarkers in ovarian cancer. The value of this tool will be in the preliminary assessment of biomarkers in ovarian cancer, particularly for research groups with limited bioinformatics facilities.

## Methods

### Gene expression data

Gene expression data sets were downloaded from the Gene Expression Omnibus (http://www.ncbi.nlm.nih.gov/geo/) in the form of raw data files, where possible. Only ovarian cancer datasets with survival information and at least 50 patients were included. In total, 2,129 samples across 14 datasets incorporating 7 different array platforms were utilised to develop the *OvMark* system. Table 1 contains a breakdown of the platforms used and the clinical information available with each dataset. Clinical data was manually checked to ensure that clinical factors are defined equally across studies. Clinical information where ambiguity occurred were excluded. In the case of the raw data for the Affymetrix datasets (.cel files), gene expression values were called using the GC robust multichip average method [14] and data were quantile normalised using the Bioconductor package, affy (http://www.bioconductor.org). For the dual-channel platforms, data were loess normalised [15] using the Bioconductor package limma. Where raw data was not available, the normalised data as published by the original authors was used. Hybridisation probes were mapped to Entrez gene IDs to gene centre the data to allow the comparison of the expression across disparate platforms [16]. The Entrez gene IDs corresponding to the array probes were obtained using Biomart [17] (http://www.biomart.org/) and the Bioconductor annotation libraries. Probes that mapped to multiple genes were filtered out. If there were multiple probes for the same gene, the probe values were averaged for that gene. This resulted in expression data for a total of 20,017 Entrez gene IDs across 2,129 samples. These 20,017 Entrez gene IDs corresponding to approximately 17,000 genes.

**Table 1:** **Summary description of clinical datasets used by** ***OvMark***

GEO accession	Ref.	Sample number	Platform type (probe number)	Mean age ± SD (Years)	Residual tumour information	Treatment Information (Pl/Ta/Neo/ Chemo)	FIGO stage					Histology			Tumour grade				Mean survival ± SD (months)	
							1	2	3	4	NA	Ser	End	NA	1	2	3	NA	DFS	OS
**GSE26712**	Bonome *et al.* [18]	185	Affymetrix U133A (~22 K)	×	Yes	×	×					×			×				×	47 ± 36
**GSE13876**	Crijns *et al.* [19]	157	Operon human v3 (~35 K)	58 ± 12	×	×	×					157	0	0	×				×	37 ± 40
**GSE14764**	Denkert *et a*l. [20]	80	Affymetrix U133A (~22 K)	×	Yes	×	8	1	69	2	0	62	6	12	3	23	54	0	×	34 ± 15
**GSE30161**	Ferris *et al*. [21]	58	Affymetrix U133Plus2 (~54 K)	63 ± 11	×	Pl/Neo/Chemo	×					47	1	10	×				24 ± 32	46 ± 36
**GSE19161**	Konstantinopoulos *et al.* [22]	61	Affymetrix 0.6 K Custom Chip (~600)	×	×	Pl/Ta	×					×			×				×	31 ± 19
**GSE19829**	Konstantinopoulos *et al.* [23]	70	Affymetrix U133Plus2 (~54 K) / Affymetrix U95v2 (~12 K)	×	×	×	×					×			×				×	39 ± 23
**GSE26193**	Mateescu *et al*. [24]	107	Affymetrix U133Plus2 (~54 K)	58 ± 11	Yes	×	×					83	8	16	7	32	68	0	50 ± 52	59 ± 51
**TCGA**	McLendon *et al*. [25, 26]	562	Affymetrix U133A (~22 K)	×	×	Chemo	×					×			×				×	33 ± 27
**GSE18520**	Mok *et al*. [27]	53	Affymetrix U133Plus2 (~54 K)	×	×	×	×					×			×				×	40 ± 41
**GSE31245**	Spentzos *et al*. [28]	57	Affymetrix U95v2 (~12 K)	×	×	×	×					×			×				×	42 ± 16
**GSE9899**	Tothill *et al.* [29]	285	Affymetrix U133Plus2 (~54 K)	60 ± 11	Yes	Pl/Ta/Neo	24	18	217	22	4	264	20	1	19	97	164	5	21 ± 18	31 ± 23
**GSE17260**	Yoshihara *et al*. [30]	110	Agilent- 4x44K (~41 K)	×	×	×	×					110	0	0	26	41	43	0	22 ± 18	36 ± 20
**GSE32062**	Yoshihara *et al.* [31]	260	Agilent- 4x44K (~41 K)	×	×	Pl/Ta	×					260	0	0	0	131	129	0	27 ± 23	45 ± 52
**In-house**	In-house Dataset	84	Affymetrix HuEx-1.0st v2	62 ± 12	Yes	Neo	4	11	56	11	0	80	1	3	7	16	61	0	19 ± 22	35 ± 26

Pl = Platinum, Ta = Taxane, Neo = Neoadjuvant Chemotherapy, Chemo = Chemotherapy, NA = Not Available, Ser = Serous, End = Endometrioid, DFS = Disease Free Survival, OS = Overall Survival, TCGA = The Cancer Genome Atlas, × = Missing Data, SD = Standard Deviation.

MiRNAs are frequently located within the introns of protein coding genes and in exons of non-coding transcripts. miRNA expression can be detected using conventional microarrays through host gene expression for intragenic miRNAs or by direct probe matching for intergenic miRNAs. 827 samples were processed on U133A Affymetrix arrays, while 531 were processed on U133plus2 Affymetrix arrays (1358 in total). U133A and U133plus2 microarrays have 22,277 probe sets in common. Using this information, it is possible to infer the expression of 341 miRNAs across 1,358 samples based on a previously published mapping of Affymetrix probesets to miRBase [32]. As with the gene centred data, this information was also combined with the available clinical data for survival analysis. This approach does not measure the expression of the miRNA directly but rather uses its host gene as a surrogate. The cancer genome atlas (TCGA) [33] provides matched Affymetrix data and miRNA data. These datasets were overlapped using probe information from [32] and correlation between the two datasets was used to identify potential promising surrogates. This approach was obviously restricted to the miRNAs/genes available on the platforms used by TCGA and the limitations of these technologies. ~60% of the miRNAs significantly correlated with their host gene expression data. This information is available in Additional file 1. As this is not a definitive assessment of the correlation between miRNAs and their host genes the *OvMark* user is not restricted from searching all 341 miRNAs but is advised to use caution.

### Survival analysis

The first stage in *OvMark* survival analysis dichotomises the expression of the gene of interest based on a median, high (within the 75% quartile) or low expression (within 25% quartile) cutoff. For example, if median expression is chosen, the expression of the gene of interest in a particular dataset is calculated. Those samples where the expression of the gene is greater than the median expression of that gene for that dataset are placed in the high expression group and those with less than median expression are placed in the low group. To account for study-to-study variation this phase is conducted separately for each of the 14 datasets. Once stratification is complete the individual datasets are combined and a global pooled survival analysis is performed to determine if the gene is associated with either OS and DFS. It is important to treat each dataset separately when determining if a sample belongs to the high or low expression groups, as the expression of the gene of interest will vary greatly across the different experiments/platforms. This approach is robust enough to detect the expression changes at low levels. A gene that goes from not detected to low expression, will “change quartiles”. In this presence or absence scenario, those samples where the gene is absent would be in the lower quartile, the “low expression cohort” and those samples where the gene is present would be in the higher quartile, the “high expression cohort” even though the absolute expression of the gene is very low. This is because though the interquartile range is low, it is still sufficient to distinguish between the two groups.

Survival curves are based on Kaplan-Meier estimates and the log-rank p-value is shown for difference in survival. Cox regression analysis is used to calculate hazard ratios. The R package survival is used to calculate and plot the Kaplan-Meier survival curve. All calculations are carried out in the R statistical environment (http://cran.r-project.org/). For further details see Madden *et al*. [5].

### Web server

The interface (that we have named *OvMark*) is available on a publically accessible a web server at http://glados.ucd.ie/OvMark/index.html and will be updated on a regular basis. The software uses Common Gateway interface (CGI) to link the web server with the R/PERL based algorithm. All calculations are carried out in real-time. All data/scripts are available upon request from the authors.

### User input options

The software incorporates the clinical data made available by the original authors. This allows the gene expression data to be analysed based on one or more common clinical parameters including patient age, residual tumour, histological type, chemotherapy, neoadjuvant chemotherapy, taxane treatment, platinum treatment, tumour grade, Federation of Gynaecologists and Obstetricians (FIGO) stage and histology subtype. The software also allows for the median expression and the upper or lower quartiles of the expression of the gene of interest to be used to determine high and low groups within each of the 14 individual datasets.

### Testing OvMark using known markers of prognosis in Ovarian cancer

*OvMark* was run using 14 previously identified prognostic markers, LPR [33], PRL [34], SPP1 [34], IGF2 [35], MIF [36], CA125 [37], BRCA1 [3], BRCA2 [3], CDKN1B [38], MLH1 [39], ApoA1 [40], SNAI2 [41], CXCL12 [42] and IFNG [43]. Each of these 14 genes was queried in our novel *OvMark* database using the median expression option to dichotomise the data and DFS and OS as the survival endpoints.

### Screen of all genes for their prognostic potential

All ~20,000 Entrez gene IDs corresponding to approximately 17,000 genes were queried in the *OvMark* database. The database was dichotomised using median gene expression and overall survival was chosen as the survival endpoint. No other software parameters were used. The resultant p-values are adjusted for multiple testing using the Benjamini-Hochberg method [44]. The significant results after adjustment for multiple testing are ranked by their hazard ratio.

To illustrate the utility of the *OvMark* system we identified the 10 genes most closely associated with survival (i.e. highest and lowest hazard ratios from Cox regression). To further demonstrate their potential as biomarkers for ovarian cancer prognosis the concordance index (C-index) was calculated for each of these genes, using the bioconductor pack survcomp [45]. The C-index is a commonly used metric for the assessment of prognostic biomarker performance and has been utilised in two recent studies focussed on ovarian cancer [46, 47]. Briefly, the C-Index, measures the ability of a particular gene’s expression levels to classify patient survival times; a C-Index = 0.5 represents random classification while a C-Index = 1 represents perfect discrimination. We also calculated the probability of observing the C-Index value for each gene at random by shuffling the survival information 10,000 times and determining the C-Index for each iteration. We determined the number of times a random C-Index was greater than or equal to the “true” C-Index to generate an empirical p-value.

## Results

In order to test the robustness of our gene-centred survival meta-analysis we used a panel of 14 known markers of prognosis in ovarian cancer. In addition we chose to screen all human genes for their prognostic potential in ovarian cancer. As there is currently no large-scale robust signature for miRNAs in ovarian cancer, we tested our approach on known individual miRNAs which have previously been shown to be prognostic markers, namely let-7f-2 [12] and mir-16-1 [13]. These miRNAs were chosen as proof of concept examples, to demonstrate the robustness of *OvMark*. Many other miRNAs are significantly associated with survival depending on the combination of clinical parameters chosen.

### Ovmark results correlate with previously identified mRNA and miRNA based biomarkers for ovarian cancer

**Figure 1:**
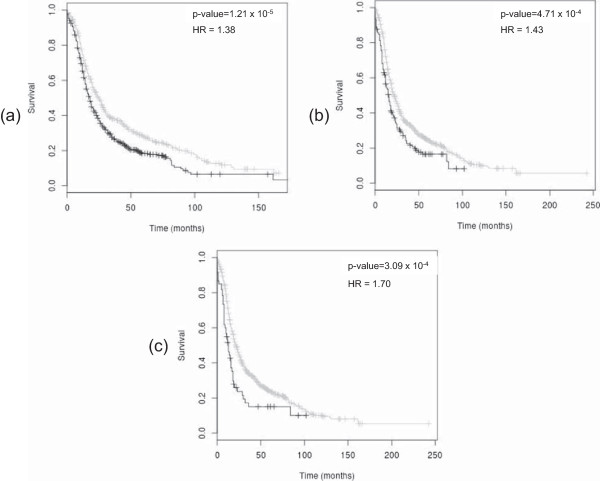
**Prognostic role of the BRCA2, PRL and CDKN1B in ovarian cancer.** In each plot black denotes high expression of the marker(s) and grey denotes low expression. **(a)** Kaplan-Meier estimates of survival, demonstrating high expression of BRCA2 is associated with poor DFS in ovarian cancer (n = 996, HR = 1.38, p = 1.21 x 10^−5^). **(b)** Kaplan-Meier estimate of survival, demonstrating that high expression of BRCA2 and PRL in combination has a greater affect on DFS than expression of either gene alone (n = 996, HR = 1.43, p = 4.71 x 10^−4^). **(c)** Combining BRCA2, PRL and CDKN1B increases the HR for DFS further i.e. grouping samples with high expression of all three genes versus the rest (HR = 1.70, p-value = 3.09 x 10^−4^, n = 996).

Each of the 14 ovarian cancer gene expression markers identified above were analysed separately within *OvMark* using median expression to dichotomise the data and DFS and OS as the survival end points. All patients in the *OvMark* database were chosen for this analysis without sub-selection based on any clinical parameters. This information is summarised in Table 2. A hazard ratio (HR) of greater than 1 indicates a negative effect on survival and a HR of less than one has a positive effect. For HRs greater that 1, the higher the HR the greater the effect the gene has on survival. For HRs less than 1, the lower the HR the greater the effect the gene has on survival. As can be seen from Table 2, several of the markers were significantly or borderline significantly associated with ovarian cancer patient outcomes, with the direction of those associations all consistent with what would be expected based on prior ovarian cancer studies. BRCA2 is one of the most significant individual marker of prognosis as can be seen in Figure 1(a) (HR for DFS = 1.38, p = 1.21 × 10^−5^, n = 996). Combining the markers can improve HRs in comparison with single markers alone. For example, the Kaplan-Meier DFS plot for BRCA2 and PRL in combination (i.e. comparing the OS of patients with greater than median expression of both BRCA2 and PRL, against the rest) is shown in Figure 1(b) (HR = 1.43, p = 4.71 × 10^−4^ n = 996). Patients with high-level expression of both BRCA2 and PRL in their ovarian cancers have a worse prognosis than those with high expression of BRCA2 or PRL alone in their ovarian cancers (with a HR of 1.43 versus a HR of 1.38 or 1.25 respectively). When greater than median expression of CDKN1B is combined with greater than median expression of BRCA2 and PRL (Figure 1(c)) the HR for OS is further increased (HR = 1.70, p = 3.09 × 10^−4^, n = 996).

**Table 2:** ***OvMark*** **results for known markers of ovarian cancer using the median cut-off**

Gene symbol	Survival endpoint	Hazard ratio	P-value	Sample number
LEPR [33]	OS/DFS	1.14/1.08	0.04/0.28	1833/996
PRL [34]	OS/DFS	1.05/1.25	0.49/6.78 × 10^−3^	1990/996
SPP1 [34]	OS/DFS	1.13/1.17	0.05/0.03	1990/996
IGF2 [35]	OS/DFS	1.22/0.99	0.06/0.99	709/422
SNAI2 [41]	OS/DFS	1.28/1.30	5.89 × 10^−5^/3.37 × 10^−4^	1990/996
CXCL12 [42]	OS/DFS	1.27/1.42	2.42 × 10^−4^/1.52 × 10^−6^	1833/996
ApoA1 [40]	OS/DFS	1.02/1.17	0.77/0.03	1990/996
MIF [36]	OS/DFS	0.89/0.89	0.09/0.13	1833/996
CA125 [37]	OS/DFS	0.89/0.97	0.09/0.69	1651/924
BRCA1 [3]	OS/DFS	1.13/1.01	0.04/0.26	1990/996
BRCA2 [3]	OS/DFS	1.18/1.38	0.01/1.21 × 10^−5^	1833/996
CDKN1B [38]	OS/DFS	1.18/1.14	9.13 × 10^−3^/0.07	1990/996
MLH1 [39]	OS/DFS	0.98/0.81	0.78/5.18 × 10^−3^	1833/996
IFNG [43]	OS/DFS	0.80/1.06	4.29 × 10^−3^/0.43	1833/996

DFS = Disease Free survival, OS = Overall Survival.

**Figure 2:**
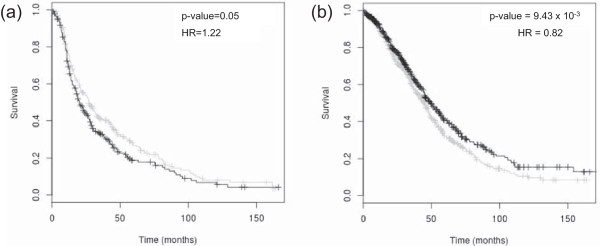
**miR-16-1 and let-7f-2 are associated with prognosis in ovarian cancer.** In each plot black denotes high expression of the miRNA and grey denotes low expression. **(a)** High miR-16-1 expression is a marker of poor prognosis in ovarian cancer using low expression to dichotomise the data and DFS as the survival endpoint (HR = 1.22, p-value = 0.05, n = 514). **(b)** High expression of let-7f-2 is associated with good prognosis in ovarian cancer using median expression to dichotomise the data and OS as the survival endpoint (HR = 0.82, p-value = 9.43 x 10^−3^, n = 1241).

The miRNA MiR-16-1 has previously been associated with poor prognosis in ovarian cancer [13] and let-7f-2 is a member of the let-7 family of miRNAs which are frequently down regulated in cancer and are associated with a good prognosis in ovarian and other cancers [48]. The expression of both these markers is significantly correlated with their host gene expression in the TCGA data. To test our approach and to demonstrate the robustness of these markers in ovarian cancer, we examined the association of the host genes of these miRNAs with prognosis in ovarian cancer using our *OvMark* database. Our results for miR-16-1 and let-7f-2 are shown in Figure 2 (a) and (b), respectively. We confirmed high expression of the host gene of miR-16-1 to be associated with a poor prognosis (HR = 1.22, p-value = 0.05, n = 514) and high expression of the host gene of let-7f-2 to be associated with a good prognosis (HR = 0.82, p-value = 9.43 × 10^−3^, n = 1241).

In summary, outcome predictions by the *OvMark* database for known ovarian cancer biomarkers are consistent with previously published data, thus validating the potential utility of this database for the study of the clinical and outcome implications of the expression of other genes and miRNAs in ovarian cancer.

### A transcriptome-wide screen using OVMARK identifies of potential novel biomarkers

**Figure 3:**
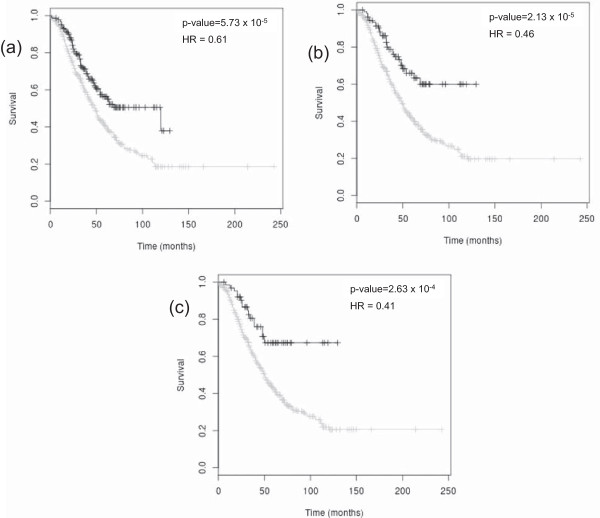
**Prognostic role of SNAI3, PCDP1 and SERPINA2 in ovarian cancer.** In each plot black denotes high expression of the marker(s) and grey denotes low expression. **(a)** Kaplan-Meier estimates of OS, demonstrating high expression of SNAI3 to be a marker of good prognosis in ovarian cancer (n = 827, HR = 0.61, p = 5.73 x 10^−5^). **(b)** Kaplan-Meier estimate of OS demonstrating that ovarian cancer samples with high expression of both PCDP1 and SNAI3 are associated with a better prognosis than ovarian cancers with high expression of either marker on its own (n = 827, HR = 0.46, p = 2.13 x 10^−5^). **(c)** Combing PCDP1, SNAI3 and SERPINA2 gives an even lower HR (HR = 0.42, p-value = 1.29 x 10^−3^, n = 827).

Although all of the prognostic markers above chosen for testing have been well studied in ovarian cancer (in some cases with mixed results), only BRCA2, SNAI2 and CXCL12 were a particularly convincing marker of outcome in *OvMark*. We then queried all human genes in *OvMark* so as to identify which genes had the greatest association with OS of ovarian cancer patients. The results for the top ten genes are summarised in Table 3, along with their C-index. Additional file 2, shows a forrest plot for each of the 10 genes to illustrate the C-index and the upper and lower bounds. The sample numbers vary depending on the number of platforms with probes for the gene of interest. The genes are ranked in Table 3 based on the strength of the OS hazard ratio. Figure 3 shows the Kaplan-Meier plot for three of genes, snail homolog 3 (SNAI3), primary ciliary dyskinesia protein 1 (PCDP1) and serpin peptidase inhibitor, clade A (alpha-1 antiproteinase, antitrypsin), member 2 (SERPINA2). Figure 3(a) shows the Kaplan-Meier plot for SNAI3 on its own (HR = 0.61, p = 5.73 × 10^−5^) and Figure 3(b) and (c) show the plots for SNAI3 in combination with PCDP1 and in combination with PCDP1 and SERPINA2, respectively. What is most striking about these results is how few of the genes have been previously linked to ovarian cancer, with only follicle stimulating hormone receptor (FSHR) having been previously well studied in ovarian cancer [49].

**Table 3:** **The top 10 highest/lowest hazard ratios from** ***OvMark*** **screen of all Entrez Gene IDs (n = 20,016) using OS as the survival end point and the median cut-off**

Gene symbol	Entrez gene ID	Previous association with ovarian cancer	Previous association with cancer	Hazard ratio	P-value*	Sample number	C-Index	P-value
SNAI3	333929	Yes [50]	Yes [51]	0.61	0.03	827	0.62	2.0 × 10^−4^
VWA3A	146177	No	No	0.63	0.02	610	0.59	2.1 × 10^−3^
DNAH12	201625	No	No	0.64	0.01	610	0.61	2.0 × 10^−4^
SERPINA2	390502	No	No	0.64	0.01	1293	0.64	< 0.0001
TMEM181	57583	No	No	1.56	0.03	527	0.60	8.0 × 10^−4^
PCDP1	200373	No	No	0.65	8.96 × 10^−3^	827	0.62	< 0.0001
ANKIB1	54467	No	No	1.53	0.04	527	0.60	3.2 × 10^−3^
C11ORF88	399949	No	No	0.66	0.01	910	0.62	< 0.0001
FSHR	2492	Yes [52]	Yes [52]	0.68	9.14 × 10^−3^	1833	0.60	1.0 × 10^−3^
TBC1D23	55773	No	Yes [53]	1.46	0.05	610	0.60	1.2 × 10^−3^

*P-value adjusted for multiple testing using the Benjamini-Hochberg method [44].

## Discussion

*OvMark* provides a user-friendly tool for examining putative prognostic biomarkers in ovarian cancer. It builds on our previous work in breast cancer where we successfully combined multiple datasets to perform cross-dataset survival analysis [5, 6]. The value of the approach used here is based on its simplicity of operation, and the statistical power gained through the combination of a large cohort of patients as compared to single microarray experiments. Unlike previous approaches [54], the *OvMark* system allows users to assess prognostic markers across multiple microarray platforms by utilising study by study dicotimisiation to reduce batch effects. We are therefore not reliant on complex dataset transformations. Also, as the database is gene-centred, rather than probe-centred, we are not limited to the gene coverage of a particular platform. In summary, *OvMark* allows the analysis of ~20,000 unique Entrez gene IDs in 2,129 ovarian cancer samples. It is our intension to increase this number as more ovarian cancer datasets become available.

Surprisingly, those genes found to have the greatest correlation with outcome across the dataset have not been studied in ovarian cancer, or in some cases in any cancer. For example, of the three genes with the greatest association with overall survival SNAI3, VWA3A and DNAH12, only SANI3 has been previously associated with carcinogenesis where it is involved in epithelial to mesenchymal transition [55].

After confirming the robustness of our algorithm using genes already identified as biomarkers in ovarian cancer we proceeded to examine its potential for inferring the prognostic ability of miRNAs from the gene expression data. The attraction of miRNA biology to cancer researchers arises from the potential of miRNAs to alter an entire pathway or indeed, pathways. miRNAs have been heavily studied in ovarian cancer; however, their role as prognostic markers are not well characterised. There are only a few large-scale studies which incorporate miRNA profiling integrated with detailed clinical data [56, 57]. Despite the huge efforts required to compile these datasets, their sample numbers are only in the hundreds and therefore they have limited statistical power. However, as shown here in our study, there is a wealth of gene expression data available with detailed clinical information which can be exploited by inferring miRNA activity from host gene expression.

Again, our algorithm gene centres the data, and allows us to examine miRNAs as prognostic markers in ovarian cancer. We were able to confirm the results of other studies [12, 13, 48], which demonstrated that reduced expression of let-7f-2 and increased expression of miR-16-1 are associated with poor prognosis in ovarian cancer. It should be noted, however, that not all miRNAs and host genes are co-expressed [9] and care needs to be taken when interpreting miRNA results from *OvMark*. This issue cannot be fully resolved until such time as there is a better understanding of which miRNAs are co-expressed with their host genes (using the TCGA data we estimate this number to be ~60%) and, further, if those that are not significantly co-expressed do so in a disease/tissue-specific manner. It also needs to be determined whether the miRNAs themselves are subject to some level of post-transcriptional regulation.

Our new algorithm *OvMark* has some limitations. It can not overcome the inherent problems associated with transcriptomic analysis of ovarian cancer, in that often samples are taken at a late stage. The identification of biomarkers through the *OvMark* system should only be considered as part of the discovery phase. In order to confirm the utility of genes identified by OvMark further validation is required to assess biomarker candidates in an independent replication cohort. The *OvMark* system will allow researchers to easily asses the prognostic performance of their targets of interest within a large scale dataset and reduce false discovery rates when prioritising putative biomarkers for subsequent validation in their laboratories.

## Conclusions

In this study, we have developed a simple user-friendly tool for examining putative gene/miRNA prognostic markers in ovarian cancer. The value of this tool is both in the simplicity of its design and the robustness of its approach. It is designed with non-bioinformatic research groups in mind and will be of great value in the preliminary assessment of putative biomarkers in ovarian cancer.

## Electronic supplementary material

Additional file 1: **miRNAs significantly correlated with their host gene expression data.** (XLSX 14 KB)

Additional file 2: **Forest plot illustrating the concordance index (CI) for each of the 10 genes most closely associated with patient outcome.** The statistical significance of each CI was demonstrated by shuffling the survival information 10,000 times and calculating an empirical p-value. (TIFF 37 KB)
