# Supplementary figures and images for: OvMark: a user-friendly system for the identification of prognostic biomarkers in publically available ovarian cancer gene expression datasets
Source: Mol Cancer. 2014 Oct 24;13:241. doi: 10.1186/1476-4598-13-241 (PMC4219121; doi:10.1186/1476-4598-13-241)

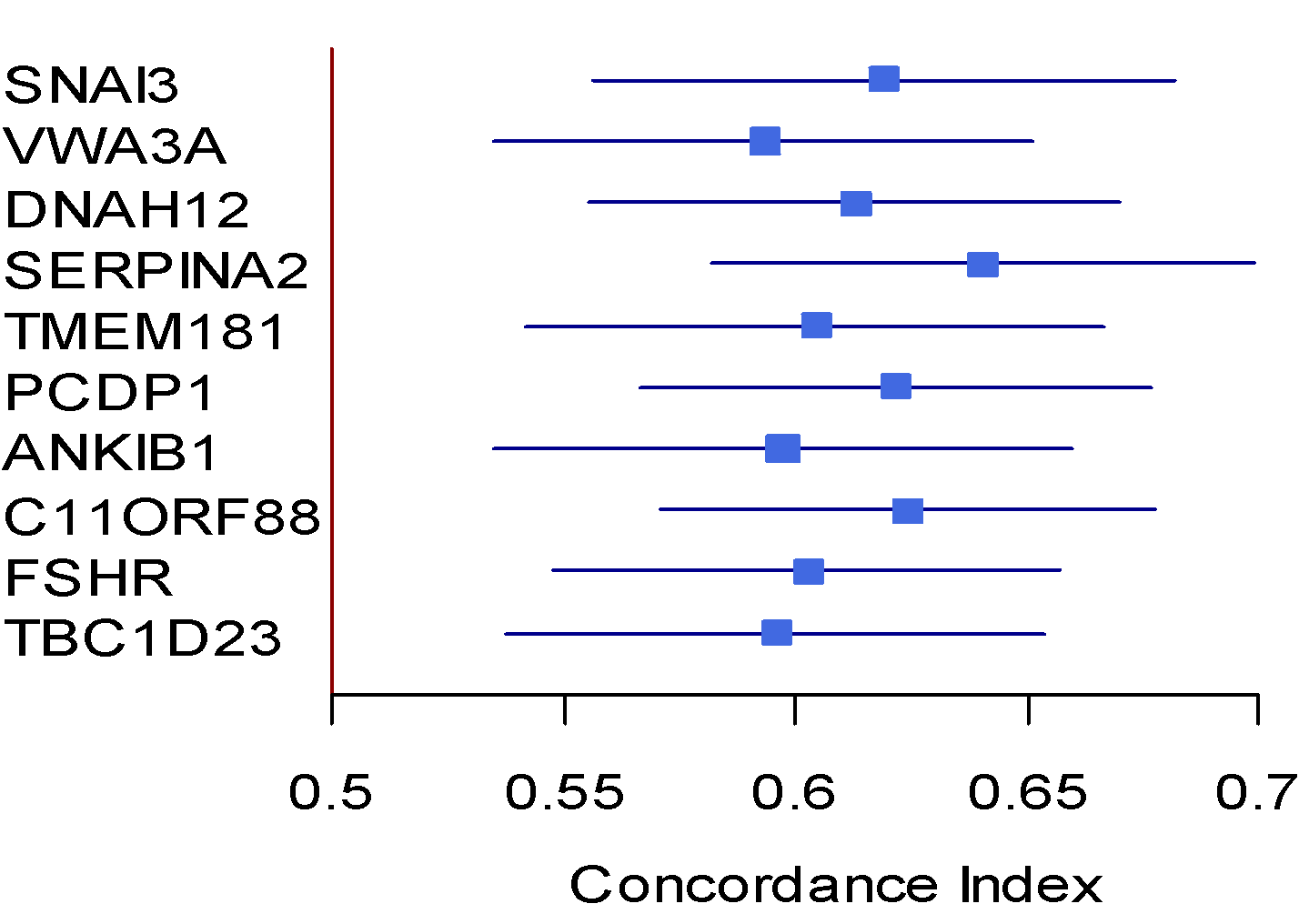

Supplement: Supplementary file 2 — Additional file 2: Forest plot illustrating the concordance index (CI) for each of the 10 genes most closely associated with patient outcome. The statistical significance of each CI was demonstrated by shuffling the survival information 10,000 times and calculating an empirical p-value. (TIFF 37 KB) [file 12943_2014_1442_MOESM2_ESM.tiff]
